# Tobacco use and perceived risk of lung cancer in a safety-net population

**DOI:** 10.21203/rs.3.rs-7802289/v1

**Published:** 2025-11-25

**Authors:** Mary E. Gwin, Heidi A. Hamann, Tanushree Prasad MA, Megan A. Mullins, Sarah T. Malone, Jennifer Rodriguez BA, Shayaan Khimani, Sheena Bhalla, Vijaya Natchimuthu, Andrea R. Semlow, Maria J. Casco, Rasmi G. Nair, Lynn N. Ibekwe-Agunanna, David H. Johnson, George Oliver, Urooj Wahid, Song Zhang, David E. Gerber

**Affiliations:** UT Southwestern Medical Center; University of Arizona; UT Southwestern Medical Center; UT Southwestern Medical Center; UT Southwestern Medical Center; UT Southwestern Medical Center; UT Southwestern Medical Center; UT Southwestern Medical Center; Parkland Health; Parkland Health; Parkland Health; UT Southwestern Medical Center; UT Southwestern Medical Center; UT Southwestern Medical Center; Parkland Center for Clinical Innovation; UT Southwestern Medical Center; UT Southwestern Medical Center; UT Southwestern Medical Center

**Keywords:** disparities, health behaviors, health literacy, lung cancer screening, safety-net, smoking cessation, tobacco use, under-represented minority

## Abstract

**Background:**

Under-represented populations may have higher smoking rates and face greater risk of lung cancer. We examined perceptions of lung cancer risk and smoking behaviors in an urban safety-net lung cancer screening (LCS) population.

**Methods:**

We conducted surveys of English- and Spanish-speaking individuals undergoing first-time low-dose computed tomography (LDCT). Current smoking was defined as one or more cigarettes within the past month. We characterized smoking behavior according to the transtheoretical model of health behavior change. Results were analyzed by Chi-square test, Fisher’s exact test, and multivariable logistic regression models.

**Results:**

Among 447 invited individuals, 411 (92%) participated in the survey, of whom 53% were Black, 18% were Hispanic, 56% reported income below the federal poverty level, 62% had graduated high school, and 79% were current smokers. Seventy percent reported some degree of worry about developing lung cancer, with 40% perceiving they were at risk in the next 10 years. In multivariable analysis, recent quit attempts were significantly associated with older age, Black race, perceived lung cancer risk in the next ten years, and level of worry about developing lung cancer. Specifically, individuals perceiving personal lung cancer risk were less likely to have made a recent quit attempt (OR 0.47; *P* = 0.04), while those reporting a lot of worry about developing lung cancer were more likely to have attempted to quit in the prior 12 months (OR 3.81; *P* = 0.001). Men (OR 1.71; *P* = 0.03) and Hispanic individuals (OR 3.87 compared to Black individuals; *P* < 0.001) were more likely to perceive personal risk of lung cancer. When grouped according to health behavior change (precontemplation/contemplation, preparation, action, maintenance), smoking behavior was not associated with level of worry about lung cancer (*P* = 0.46).

**Conclusions:**

In an urban, safety-net LCS population, current smoking rates are high and perceived lung cancer risk varies by numerous demographic characteristics. While most individuals reported worry about lung cancer, which correlated with past quit attempts, this concern is not associated with overall current smoking behavior. Given disparities in smoking rates and lung cancer risk, a nuanced understanding of factors affecting smoking behaviors may optimize cessation interventions in under-represented populations.

## Introduction

As awareness of the detrimental effects of smoking has grown, smoking rates in the United States (U.S.) have fallen substantially. Currently, it is estimated that 12% of U.S. adults smoke cigarettes, compared to approximately one-third of women and more than 55% of men in the mid-1960s.^1,2^ This prevalence is not distributed equally, as men, residents of midwestern and southern states, and lower socioeconomic status (SES) individuals have higher current smoking rates.^3,4^ Furthermore, the decline in smoking rates and cigarette consumption has been significantly greater for White individuals than for Black individuals. At the same time, there are also disparities in the health consequences of smoking. Women and non-White individuals develop lung cancer after less smoking than do men and White populations.^5^ Smoking may also disproportionately increase the risk of cardiovascular disease in Black persons compared to other groups.^6^

Although smoking cessation confers numerous health benefits—including decreased risk of multiple types of cancer, cardiovascular disease, and chronic obstructive pulmonary disease^7^—it remains a challenging goal. Fewer than 10% of people who use tobacco successfully quit smoking in a given year, even though two-thirds express wanting to quit and more than half make at least one quit attempt.^8^ Cessation efforts and success also vary across populations. Compared to non-Hispanic Whites, Black individuals tend to smoke longer before attempting to quit, make more quit attempts, but are less likely to quit.^9,10^ These disparities have grown over time, with quit rates increasing significantly more in White populations than they have among Black individuals with tobacco use.^11^ While pharmacologic, non-pharmacologic, and multimodal interventions have demonstrated effectiveness in achieving tobacco abstinence, these resources are used less frequently by non-White groups and those with lower SES.^12,13^

Lung cancer screening represents a key teachable moment for smoking cessation.^14,15^ Indeed, counseling on continued abstinence or smoking cessation represents a required component of shared decision-making for lung cancer screening.^16^ In this study, we evaluated the association between perceived lung cancer risk perceptions and smoking behaviors, hypothesizing that individuals perceiving greater risk would be more likely to exhibit smoking cessation behaviors.^17^ This trend was observed in the National Lung Screening Trial (NLST),^18^ but the NLST population was younger, healthier, more educated, and less racially diverse than the real-world screening-eligible population.^19^ To gain insight into health beliefs and smoking behaviors in a contemporary, diverse population, we studied individuals referred for lung cancer screening in an urban, safety-net healthcare system.

## Methods

### Study setting

This study was approved by the UT Southwestern Institutional Review Board (STU-2019–1388). Participants provided informed consent prior to undergoing study procedures, including survey participation. We identified patients initiating lung cancer screening at Parkland Health (Parkland), the integrated safety-net healthcare provider of Dallas County, Texas. Dallas County has a population of 2.6 million (42% Hispanic, 22% Black), of whom 14% live in poverty and 21% lack health insurance coverage.^20^ Compared to populations included in major lung cancer screening trials, the population served by the Parkland lung cancer screening program includes a higher proportion of under-represented minorities, people with current tobacco use, and individuals with moderate or severe comorbidity burden.^21^

### Data collection

English- and Spanish-speaking individuals undergoing first-time low-dose computed tomography (LDCT) for lung cancer screening were eligible to participate in a survey assessing demographic information (age, sex, race/ethnicity, marital status, educational background, household income), self-described health literacy and quality of health, detailed smoking behaviors (duration and volume of tobacco use, age of first tobacco exposure, previous quit attempts, time since last cigarette), intentions for smoking cessation (quit attempts in the last year, confidence in ability to quit smoking permanently and completely, openness to using resources including counseling, medications, and/or smoking cessation programs), perceived lung cancer risk, and knowledge and perceptions of lung cancer screening. Current tobacco use was defined as smoking one or more cigarettes within the past month.^22^

Survey items (see **Supplemental Materials**) were available in either English or Spanish. Items that did not already have validated Spanish translations were evaluated for conceptual equivalency through the UT Southwestern Language Validation Resource.

Individuals enrolled in a study of lung cancer screening navigation intervention from February 2017 to February 2019^29^ were invited to participate in the survey. Consecutive patients referred for lung cancer screening who spoke English or Spanish were eligible for the navigation trial. All enrolled persons, whether ultimately assigned to the navigation intervention (additional phone calls to remind patients about their scheduled LDCT and other screening-related appointments) or to usual care, were offered the opportunity to take the survey. Surveys were performed after ordering (generally by primary care clinicians) but before performance of the first LDCT and before randomization to navigation or usual care. The surveys generally took 20–30 minutes to complete and were conducted by telephone, with study staff recording participant responses in a Research Electronic Data Capture (REDCap) database, and. Individuals who completed the survey received a $15 gift card as recognition for their time and effort.

### Statistical analysis

Demographic data were described as frequencies with percentages. Smoking history characteristics were described as medians with interquartile ranges. We used the Chi-square test or Fisher’s exact test to compare patient characteristics according to smoking cessation behaviors. Variables with *P* values < 0.1 were included in multivariable logistic regression models to calculate odds ratios and 95% confidence intervals for outcomes of smoking cessation behaviors and perceived lung cancer risk. The level of statistical significance was set to *P* < 0.05. All statistical analyses were performed using SAS 9.4 (SAS Institute Inc., Cary, NC USA).

## Results

Among 447 individuals invited, 411 (92%) participated in the survey, of whom 90% completed all survey questions. Seventy-six percent of participants were under age 65 years, 53% were Black, 18% were Hispanic, 56% reported income below the federal poverty level, and 62% had graduated high school. Seventy percent rated their health literacy “good” or “very good”; 70% reported some degree of worry about developing lung cancer, with 40% perceiving they were at risk in the next 10 years. Additional respondent characteristics and smoking behaviors are shown in Table 1. Median age of first cigarette was 16 years, median smoking history was 40 pack-years, and 65% of individuals reported smoking within the past day. Nearly half (45%) had a successful prior quit attempt lasting one year or more; however, only 16% of all respondents reported that their most recent cigarette was at least one year prior to the survey.

Table 2 displays respondent characteristics associated with smoking quit attempts in the last 12 months. For this analysis, only respondents (N=324, 79%) with current tobacco use (defined as those who last smoked one or more cigarettes within one month prior to the survey) were included. Overall, 205 individuals (63%) reported a quit attempt, with attempts significantly more common for older (*P*=0.01) and for Black (*P*=0.04) respondents. We observed near significant associations between cessation attempts and both perceived lung cancer risk and level of worry about developing lung cancer. Notably, individuals with *perceived risk* of lung cancer were less likely to have prior quit attempts (58% vs. 68%; *P*=0.06), while those with the greatest *worry* about lung cancer were more likely to have attempted to quit: 75% of those reporting a lot of worry, 59% of those with a little worry, 60% with none (*P*=0.07). In multivariable analysis (Table 2), recent quit attempts remained significantly associated with older age, Black race, perceived lung cancer risk in the next ten years, and level of worry about developing lung cancer. Specifically, individuals perceiving lung cancer risk were less likely to have made a recent quit attempt (OR 0.47; *P*=0.04), while those reporting a lot of worry about developing lung cancer were more likely to have attempted to quit in the prior 12 months (OR 3.81; *P*=0.001).

We also asked participants with current tobacco use about their anticipated future smoking behaviors, including the likelihood of quitting permanently, seeking counseling/support to help quit smoking, and enrolling in a smoking cessation program (Table 3). Overall, 246 of respondents (76%) felt they will quit permanently; 201 (63%) would seek counseling/support; 200 (62%) would enroll in a cessation program. Compared to White individuals, Black individuals were significantly more likely to predict permanent cessation (81% versus 64%; *P*=0.006) and to seek counseling/support (72% versus 46%; *P*<0.001). Hispanic respondents were the most likely to predict cessation (86%). Younger respondents were more likely to consider enrolling in a smoking cessation program (*P*=0.004).

Among respondents, 187 (48%) perceived a lifetime risk of lung cancer, which was significantly associated with sex, race/ethnicity, education level, and health literacy (Table 4). In multivariable analysis, men (OR 1.71; *P*=0.03) and Hispanic individuals (OR 3.87 compared to Black individuals; P<0.001) were more likely to perceive such risk. Interestingly, in univariate analysis, perceived risk of lung cancer was also more common among individuals with lower education level and lower health literacy. Specifically, 55% of individuals without a high school diploma felt they were at risk for lung cancer, compared to 48% of high school graduates and 42% of those with post-secondary education (P=0.03), although the association did not retain significance in the multivariable model (*P*=0.56 and 0.13). Among participants who rated their health literacy as okay/poor/very poor, 55% felt they were at risk, compared to 43% who rated their health literacy as very good/good (P=0.03), but there was no significant association in multivariable testing (*P*=0.55).

**Figure 1:**
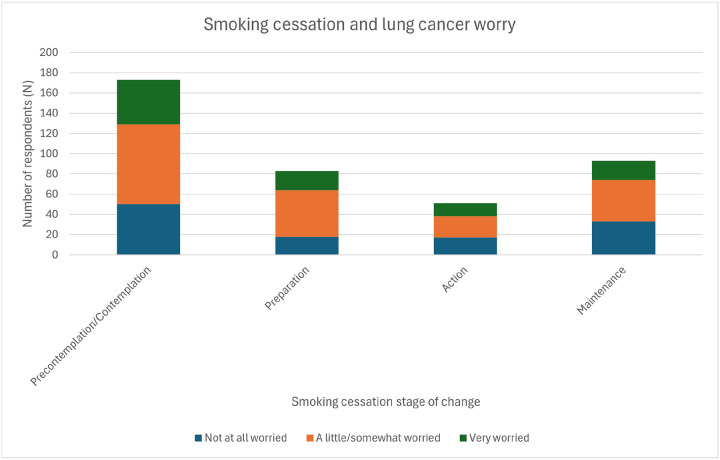
Association between smoking behavior (according to the transtheoretical model of health behavior change) and lung cancer worry (*P*=0.46).

Respondent level of worry about getting lung cancer was not associated with smoking behavior broadly characterized according to the transtheoretical model of health behavior change^30^ (*P*=0.46). In Figure 1, respondents with no specific plans to quit smoking were grouped in the “precontemplation/contemplation” phase, those planning to quit within 6 months in the “preparation” phase, those already cutting down on their smoking in the “action” phase, and those who had already quit in the “maintenance” phase.

## Discussion

Under-represented minority and lower socioeconomic status populations represent vulnerable groups that may have higher smoking rates and greater likelihood of smoking-related morbidity, including lung cancer. Because smoking remains the leading cause of preventable death worldwide^31^ and markedly increases risk across a spectrum of lung disease (from chronic obstructive and interstitial lung diseases to acute lung injury and infections),^32^ we examined detailed smoking behaviors and health-related beliefs in an urban safety-net population. In this study of more than 400 individuals, we identified numerous associations between respondent characteristics, smoking behavior, and perceived lung cancer risk. However, worry about lung cancer did not correlate with current or planned efforts to quit smoking.

The racial and ethnic composition of our study population (more than 80% under-represented minorities) stands in clear contrast to most studies in this area, which either do not report these characteristics or have overwhelmingly (> 75%) non-Hispanic white populations.^33,34^ This diversity provides an important opportunity to evaluate health beliefs and smoking behaviors in non-White groups. Interestingly, we found that Hispanic individuals perceived the greatest risk of lung cancer, while Black respondents reported the most prior and planned attempts to quit smoking. The elevated lung cancer risk perception among Hispanic respondents could, perhaps, be one of many factors driving lower rates of current smoking observed among this demographic group.^1^ Two studies conducted among a subset of participants enrolled in the NLST, less than 10% of whom were Black, found that Black individuals had lower perceived risk of smoking-related diseases compared to White individuals, lower confidence that they could quit smoking, and were less likely to make a quit attempt over a five- to six-year period of follow-up.^35,36^ However, in a community-based study of more than 500 individuals, Black individuals reported greater confidence in the ability to quit smoking than did White individuals.^37^ Our study’s findings echo the latter report, with Black respondents demonstrating both higher confidence about eventual smoking cessation and higher rates of intended adoption of cessation-related resources. Conflicting conclusions about the relationship between race/ethnicity and attitudes toward smoking cessation suggest that additional research is needed to better understand this nuanced topic.

Notably, we also observed higher perceived risk of lung cancer among individuals with lower education level and lower self-reported health literacy. To our knowledge, this is the first study to report such findings for lung cancer, but they appear to mirror observations among patients undergoing other types of cancer screening. In a cross-sectional analysis of more than 800 women undergoing screening mammography, lower health literacy was associated with overestimation of individual breast cancer risk.^38^ A systematic review of colorectal cancer screening participants found either an inverse relationship between health literacy and informed decision-making about screening, or no association between these factors.^39^ We also observed that men were significantly more likely to perceive themselves at risk of lung cancer, a trend possibly driven by higher rates of smoking among men compared to women.^40^ However, these discrepancies in perceived lung cancer risk do not reflect clinical reality: lung cancer is diagnosed at similar rates in both sexes, and has remained the leading cause of cancer-related death among women since the late 1980s.^41^ Therefore, clinicians may need to be particularly vigilant about counseling female patients about lung cancer risk to promote and achieve gender equity in lung cancer screening and smoking cessation.

Surprisingly, perceived risk of lung cancer correlated neither with motivation to quit smoking nor with smoking status. This observation may reflect the numerous and complex factors underlying behavior change, including mental health, financial and social circumstances, and self-efficacy.^42,43^ A qualitative health survey of 1,205 individuals from low-income neighborhoods (61% Black, 20% Latino) found that access to affordable cessation aids and support services; encouragement from healthcare providers, friends, and family; and the potential financial savings from not purchasing tobacco products drove patient motivation for smoking cessation.^44^ These considerations relate more to practical considerations than to the abstract notion of long-term risk reduction. Additionally, we observed a complex relationship between perceptions of lung cancer risk and *past* quit attempts. Individuals who felt they had the greatest *risk* of developing lung cancer were less likely to have attempted tobacco cessation. In these cases, behavior may have driven concerns. That is, respondents may have felt that their inability or unwillingness to try to quit smoking placed them at heightened risk for future malignancy. Conversely, those who reported the greatest *worry* about lung cancer were more likely to have past quit attempts. Here, concerns may have driven behavior. Among a subset of NLST participants, those with prior tobacco use had lower perceived risk of lung cancer compared to individuals with ongoing tobacco use.^45^ In our study, we observed no difference in perceived risk based on current or former smoking status, although we may be underpowered to detect a difference given a much higher proportion of respondents with ongoing tobacco use compared to the NLST.

Despite the distinct demographic features of our study population, reported smoking behaviors resembled those reported in the broader U.S. population, with both first cigarette use and onset of regular smoking occurring in the mid-teens.^46^ Most individuals with tobacco use require multiple attempts to quit smoking successfully,^47^ a trend also apparent in our study population. While nearly half of respondents reported a previous period of quitting for a year or more (and almost two-thirds had tried to quit within the prior year), only one-third of these continued to abstain from smoking at the time of the survey, reflecting a high rate of relapsing to active smoking. Indeed, individuals with lower SES have been shown to face heightened challenges throughout the stages of tobacco cessation, from identifying the initial need to quit to avoiding relapse.^48^ Although difficulties with accessing and affording resources, such as counseling and medications, present barriers for smoking cessation, Medicaid expansion has not improved quit rates among low-income current smokers.^49^

Lung cancer screening presents a key opportunity to address and pursue smoking cessation, although screening implementation remains low nationwide and particularly so in the southern United States, where the current study was conducted.^50,51^ Individuals who pursue lung cancer screening tend to have lower rates of current smoking and more frequent quit attempts.^52,53^ In the NLST, abnormal screening results—which occurred in about 40% of participants over time^26^—correlated with future smoking cessation.^54^ Among NLST participants, those with greater nicotine dependence were less likely to successfully quit smoking, more likely to develop lung cancer, and had higher rates of all-cause and lung cancer-specific mortality.^55^ Whether these findings translate to a diverse, real-world population such as that in the present study, which featured a far higher proportion of individuals with ongoing tobacco use than did the NLST, is not yet known.

Limitations of the current study include the single-center setting, lack of longitudinal data to track smoking behaviors over time, and restriction to English- and Spanish-speaking individuals. Strengths include the large proportion of non-White and low SES individuals (groups that have been under-represented in most smoking behaviors studies), detailed characterization of smoking history, use of validated measures, and a participation rate exceeding 90%, of whom 90% completed the entire survey.

In an urban safety-net population undergoing lung cancer screening, current smoking rates are high, past quit attempts frequent, and worry about lung cancer common. However, perceived risk of lung cancer does not correlate with previously achieved or planned smoking cessation. Identifying individuals ready to quit smoking may improve resource allocation and efficacy of cessation interventions. Ultimately, a more nuanced understanding of smoking behaviors among a diverse lung cancer screening population may enable clinicians to optimize smoking cessation support among high-risk populations in the future.

## Supplementary Material

Supplementary Files

This is a list of supplementary files associated with this preprint. Click to download.

- BMCPublicHealthsmokingbehaviorssupplementalmaterial.pdf

## Figures and Tables

**Table 1.** Respondent Characteristics and Baseline Smoking Behaviors (N = 411)

Characteristic	n (%)* or median (IQR)
**Age (y)**	
<65	307 (76)
≥65	97 (24)
**Sex**	
Female	186 (45)
Male	225 (55)
**Race/ethnicity**	
Non-Hispanic Black	217 (53)
Non-Hispanic White	118 (29)
Non-Hispanic other race	20 (5)
Hispanic (any race)	56 (14)
**Education**	
Less than high school diploma	141 (34)
High school diploma	149 (36)
Additional vocational or higher education	121 (29)
**Health literacy (self-rated)**	
Okay/poor/very poor	124 (30)
Very good/good	287 (70)
**Marital status**	
Never married	80 (20)
Married or living as married	88 (22)
Widowed/separated/divorced	238 (59)
**Annual household income**	
< $15,000	239 (58)
≥ $15,000	131 (32)
Don’t know/not sure	41 (10)
**Quality of health (self-rated)**	
Poor/very poor	90 (22)
Good/fair	266 (65)
Excellent/very good	31 (8)
Don’t know/not sure	24 (6)
**Level of worry about developing lung cancer**	
Not at all	120 (30)
A little/somewhat	188 (47)
Very worried	95 (24)
**Perceived lung cancer risk in next ten years** ^†^	
Lower perceived risk	243 (60)
Higher perceived risk	160 (40)
**Age at first tobacco use, years**	15 (13–17)
**Age when individual began smoking regularly, years**	17 (15–20)
**Total duration of smoking, years**	40 (35–45)
**Average number of cigarettes per day, n**	10 (6–20)
**Time since last cigarette**	
Same day	265 (65)
1–7 days ago	52 (13)
Less than 1 month ago	7 (2)
1 month to 1 year ago	19 (5)
More than 1 year ago	64 (16)
Don’t know/don’t remember	2 (1)
**Previous quit attempt lasting ≥ 1 year?**	
Yes	184 (45)
No	225 (55)

IQR = interquartile range

* Occasionally a respondent declined to answer a question, so numbers in each demographic subgroup may not total 411 in all cases. Percentages are rounded to the nearest whole percentage.

† Here, “lower perceived risk” denotes a response of “disagree” or “strongly disagree” to the statement “It is likely that I will get lung cancer in the next ten years,” while “higher perceived risk” denotes a response of “agree” or “strongly agree” to the same statement.

**Table 2.** Characteristics Associated with Quit Attempts in the Past 12 Months Among Individuals with Current Tobacco Use (N = 324)

Respondent Characteristic	Quit Attempt in Past 12 Months		*P* value	Multivariable analysis		
	Yes N=205 n (%)	No N=119 n (%)		OR	95% CI	*P* value
**Age**			0.01			
<65 years	147 (73)	100 (85)		Ref		
≥65 years	55 (27)	18 (15)		2.83	1.47–5.44	0.002
**Sex**			0.34			
Female	99 (48)	51 (43)				
Male	106 (52)	68 (57)				
**Race/ethnicity**			0.04			
Non-Hispanic Black	121 (59)	57 (48)		Ref		
Non-Hispanic White	48 (23)	46 (39)		0.47	0.27–0.82	0.008
Non-Hispanic other race	10 (5)	4 (3)		1.25	0.35–4.45	0.73
Hispanic (any race)	26 (13)	12 (10)		1.19	0.51–2.8	0.69
**Education**			0.78			
Less than high school diploma	68 (33)	38 (32)				
High school diploma	79 (39)	43 (36)				
Additional vocational or higher education	58 (28)	38 (32)				
**Health literacy (self-rated)**			0.53			
Okay/poor/very poor	57 (28)	37 (31)				
Very good/good	148 (72)	82 (69)				
**Marital status**			0.22			
Never married	35 (17)	29 (25)				
Married or living as married	43 (21)	26 (22)				
Widowed/separated/divorced	126 (62)	63 (53)				
**Annual household income**			0.61			
< $15,000	123 (60)	65 (55)				
≥ $15,000	62 (30)	42 (35)				
Don’t know/not sure	20 (10)	12 (10)				
**Quality of health (self-rated)**			0.07			
Poor/very poor	47 (23)	25 (21)		Ref		
Good/fair	127 (62)	78 (66)		0.83	0.46–1.52	0.55
Excellent/very good	15 (7)	14 (12)		0.62	0.24–1.58	0.31
Don’t know/not sure	16 (8)	2 (2)		3.26	0.66–16.18	0.15
**Perceived lung cancer risk (lifetime)**			0.06			
Disagree or strongly disagree	116 (57)	54 (46)		Ref		
Agree or strongly agree	87 (43)	63 (54)		0.65	0.39–1.27	0.20
**Perceived lung cancer risk (next 10 years)**			0.08			
Disagree or strongly disagree	126 (62)	61 (52)		Ref		
Agree or strongly agree	77 (34)	56 (48)		0.47	0.23–0.95	0.04
**Level of worry about developing lung cancer**			0.07			
Not at all	52 (26)	34 (29)		Ref		
A little/somewhat	94 (47)	64 (55)		1.33	0.74–2.41	0.34
A lot	56 (28)	19 (16)		3.81	1.70–8.55	0.001

**Table 3.** Characteristics associated with smoking cessation behaviors among individuals with current tobacco use.

Respondent Characteristic	Will quit permanently			Will seek counseling/support			Will enroll in cessation program		
	No* N=77 n (%)	Yes^†^ N=246 n (%)	*P* value	No* N=120 n (%)	Yes^†^ N=201 n (%)	*P* value	No* N=124 n (%)	Yes^†^ N=200 n (%)	*P* value
**Age (y)**			0.45			0.18			0.004
<65	61 (80)	185 (76)		87 (73)	160 (80)		83 (69)	164 (82)	
≥65	15 (20)	58 (24)		32 (27)	41 (20)		38 (31)	35 (18)	
**Sex**			0.51			0.43			0.72
Female	33 (43)	116 (47)		59 (49)	91 (56)		59 (48)	91 (46)	
Male	44 (57)	130 (53)		61 (51)	113 (55)		65 (52)	109 (55)	
**Race/ethnicity**			0.006			<0.001			0.41
Non-Hispanic Black	34 (44)	144 (59)		50 (42)	128 (63)		62 (50)	116 (58)	
Non-Hispanic White	34 (44)	60 (24)		51 (43)	43 (21)		41 (33)	53 (27)	
Non-Hispanic other race	4 (5)	10 (4)		6 (5)	8 (4)		7 (6)	7 (4)	
Hispanic (any race)	5 (7)	32 (13)		13 (11)	25 (12)		14 (11)	24 (12)	
**Education**			0.88			0.51			0.37
Less than high school diploma	24 (31)	81 (33)		41 (34)	65 (32)		40 (32)	66 (33)	
High school diploma	31 (40)	91 (37)		48 (40)	74 (36)		52 (42)	70 (35)	
Additional vocational or higher education	22 (29)	74 (30)		31 (26)	65 (32)		32 (26)	64 (32)	
**Health literacy (self-rated)**			0.87			0.14			0.45
Okay/poor/very poor	23 (30)	71 (29)		29 (24)	65 (32)		39 (31)	55 (28)	
Very good/good	54 (70)	175 (71)		91 (76)	139 (68)		85 (69)	145 (73)	
**Marital status**			0.61			0.56			0.90
Never married	16 (21)	48 (20)		27 (23)	37 (18)		24 (20)	40 (21)	
Married or living as married	13 (17)	55 (23)		23 (19)	46 (23)		28 (23)	41 (21)	
Widowed/separated/divorced	47 (62)	142 (58)		69 (58)	120 (59)		71 (58)	118 (59)	
**Annual household income**			0.58			0.81			0.33
< $15,000	43 (56)	144 (59)		67 (56)	121 (59)		66 (53)	122 (61)	
≥ $15,000	28 (36)	76 (31)		40 (33)	64 (31)		43 (35)	61 (31)	
Don’t know/not sure	6 (8)	26 (11)		13 (11)	19 (9)		15 (12)	17 (9)	
**Quality of health (self-rated)**			0.77			0.38			0.42
Poor/very poor	20 (26)	52 (21)		22 (18)	50 (25)		23 (19)	49 (25)	
Good/fair	45 (58)	159 (65)		83 (69)	122 (60)		84 (68)	121 (61)	
Excellent/very good	7 (9)	22 (9)		10 (8)	19 (9)		12 (10)	17 (9)	
Don’t know/not sure	5 (7)	13 (5)		5 (4)	13 (6)		5 (4)	13 (7)	

Cell percentages may not total 100 due to rounding.

* “No” indicates an answer of either “definitely will not” or “probably will not”

† “Yes” indicates an answer of either “maybe will” or “definitely will”

**Table 4.** Characteristics Associated with Perceived Lifetime Lung Cancer Risk

Respondent Characteristic	Perceived Lifetime Lung Cancer Risk		*P* value	Multivariable analysis		
	Agree or strongly agree N=187 n (%)	Disagree or strongly disagree N=206 n (%)		OR	95% CI	*P* value
**Age (y)**			0.15			
<65	136 (73)	171 (79)				
≥65	50 (27)	45 (21)				
**Sex**			0.01			
Female	70 (37)	110 (51)		Ref		
Male	117 (63)	106 (49)		1.61	1.06–2.45	0.03
**Race/ethnicity**			<0.001			
Non-Hispanic Black	84 (45)	130 (60)		Ref		
Non-Hispanic White	54 (29)	63 (29)		1.42	0.89–2.27	0.15
Non-Hispanic other race	8 (4)	8 (4)		1.67	0.59–4.72	0.33
Hispanic (any race)	41 (22)	15 (7)		3.87	1.96–7.63	<0.001
**Education**			0.03			
Less than high school diploma	76 (41)	62 (29)		Ref		
High school diploma	64 (34)	80 (37)		0.86	0.52–1.43	0.56
Additional vocational or higher education	47 (25)	74 (34)		0.65	0.37–1.13	0.13
**Health literacy (self-rated)**			0.03			
Okay/poor/very poor	65 (35)	54 (25)		Ref		
Very good/good	122 (65)	162 (75)		1.16	0.71–1.90	0.55
**Marital status**			0.23			
Never married	35 (19)	44 (21)				
Married or living as married	48 (26)	40 (19)				
Widowed/separated/divorced	103 (55)	130 (61)				
**Annual household income**			0.53			
< $15,000	112 (60)	123 (57)				
≥ $15,000	55 (29)	74 (34)				
Don’t know/not sure	20 (11)	19 (9)				
**Quality of health (self-rated)**			0.46			
Poor/very poor	46 (25)	44 (20)				
Good/fair	122 (65)	140 (65)				
Excellent/very good	12 (6)	19 (9)				
Don’t know/not sure	7 (4)	13 (6)				
**Smoking status** *			0.70			
Current tobacco use	150 (81)	170 (79)				
Former tobacco use	36 (19)	45 (21)				

* Current tobacco use is defined as smoking one or more cigarettes within the past month.

## Data Availability

Data is available upon reasonable request to the authors by contacting the corresponding author, Dr. David Gerber (david.gerber@utsouthwestern.edu).
